# The Content of Copper and Molybdenum in the Liver, Kidneys, and Skeletal Muscles of Elk (*Alces alces*) from North-Eastern Poland

**DOI:** 10.1007/s12011-015-0430-4

**Published:** 2015-07-11

**Authors:** Michał Skibniewski, Ewa M. Skibniewska, Tadeusz Kośla, Katarzyna Olbrych

**Affiliations:** Faculty of Veterinary Medicine, Department of Morphological Sciences, Warsaw University of Life Sciences-SGGW, Nowoursynowska 159 C, 02-776 Warsaw, Poland; Faculty of Animal Sciences, Department of Animal Environment Biology, Warsaw University of Life Sciences-SGGW, Ciszewskiego 8, 02-786 Warsaw, Poland

**Keywords:** Elk (*Alces alces*), Copper, Molybdenum, Liver, Kidneys, Muscles

## Abstract

The aim of the study was to evaluate the content of Cu and Mo in the liver, kidneys, and skeletal muscles of elks from north-eastern Poland. The investigation material comprised samples obtained in 2010 from 35 animals. Animals were grouped according to age (elks up to 2 years and over than 3 years). The metal concentrations were determined using coupled plasma-mass spectrometry (ICP-MS) method. The mean Cu concentrations in the liver, kidneys, and skeletal muscles were 23.08, 5.03, and 2.36 mg∙kg^−1^ wet weight, respectively. The mean Mo content in the examined samples was as follows: 0.92, 0.42, and 0.05 mg∙kg^−1^ wet weight (w.w.) in the liver, kidneys, and muscles. In the analysis of correlation between the Cu and Mo levels in particular organs, the presence of significant dependence (*p* ≤ 0.05) was observed in the liver of animals studied. The mean Cu content in the liver of animals studied is lower compared with data reported from Sweden, Russia, and North America. Concentrations of Cu and Mo in the kidneys and skeletal muscles of Polish elks are similar to data noted in healthy animals from Scandinavian region. The results suggest that elks from north-eastern Poland may be threatened by primary Cu deficiency.

## Introduction

Studies on the content of copper (Cu) and molybdenum (Mo) in the internal organs of ruminants pertain mainly to domesticated animals. There are few publications devoted to the content of these metals in the tissues of representatives of *Cervidae* family free living in the north-eastern Poland. These publications concern metal concentrations in the parenchymatous organs of the red deer (*Cervus elaphus*), which belongs to game animal whose meat is a component of the human diet [1, 2]. In the literature, there is no respective information on metal concentrations in the tissues of elk (*Alces alces*) from Polish territory. Available data for representatives of this species are from Scandinavian countries and from North America. Performed studies indicate that analysis of certain metals concentrations in the elk’s tissues (the largest representative of currently living *Cervidae*) may be a valuable source of information on its habitat. The animal lives a relatively settled way of life, and usually, its migrations do not exceed 50–80 km [3]. Elk in Poland is a game animal with the year-round protection season. The Minister of Environment issued a moratorium on shooting elk in 2001. This allowed for increasing elk’s population in Poland from 2.1 thousands in year 2000 to 8.4 in year 2010 [4]. More than 70 % of the elk’s population in Poland inhabits the north-eastern part of the country, which is a region of low industrialization with many protected areas [5].

In the mid-1980s, a disease not described before was observed in elk from south-western Sweden. The number of dead animals or shot due to the disease symptoms was 150 per year, which made 3 % of elk population in the region [6]. Performed studies revealed that the most probable reason of this “mysterious” disease was Cu deficiency and molybdenosis [3, 6, 7]. As an essential component of numerous proteins and enzymes, Cu is involved in basic vital processes such as the following: mitochondrial respiration, antioxidative activity, synthesis of neurotransmitters, and metabolism of glucose as well as lipids. Thus, its deficiency may cause acute metabolic disorders leading to many diseases or even death. Despite its important role in metabolic processes, high tissue Cu concentrations show a toxic effect on the organism. It is evident that the Cu metabolism in ruminants is influenced by interactions with different elements. The most important are Mo and S, which during digestion in the forestomachs, especially in rumen, can form thiomolybdates reducing absorption of Cu. Although Mo is essential for metabolic processes, its adverse effects can be observed even at moderately increased tissue concentrations leading to inhibition of Cu-containing enzymes. Thus, the quantitative relationship between Cu and Mo are critical for ruminants [8, 9].

This study was aimed at assessing the content of Cu and Mo in the liver, kidneys, and skeletal muscles of elks from north-eastern Poland, at finding whether mineral supply of the populations of elk in Poland differs from that in other European regions and at answering the question if there is a risk of disease observed in Swedish elk. Although the elk’s population in Poland is increasing, which may indicate a good environmental condition, their mineral status is not completely known. Therefore, monitoring of Cu and Mo content in elk tissues is important since both metals are involved in numerous metabolic functions.

## Material and Methods

**Fig. 1:**
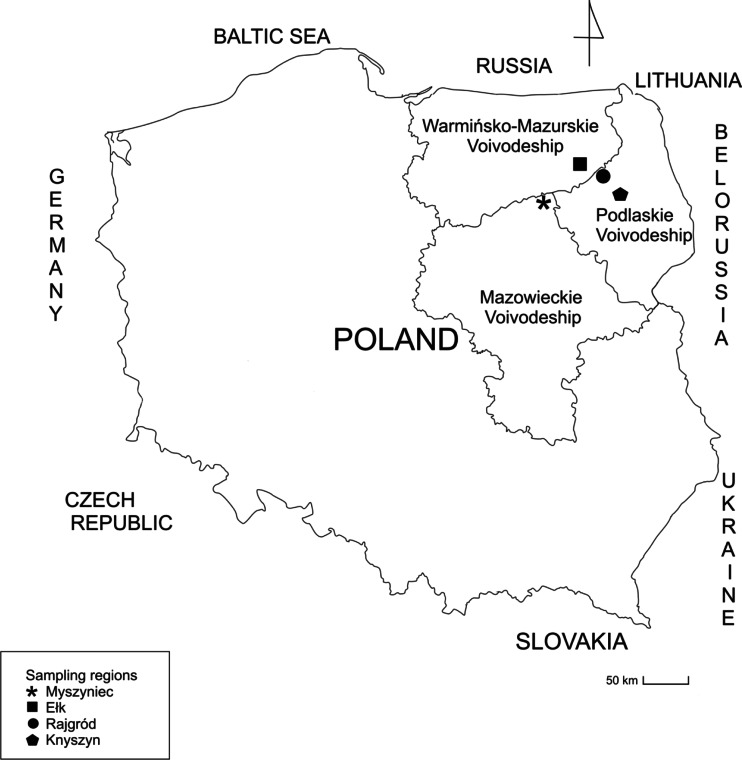
Sampling sites according to geographical locations

Study material consisted of kidney, liver, and muscle samples collected from 35 elks during a culling performed based on a permit issued by the Minister of Environment in 2010 (decision number DL.gł-6713-5/45392/10/PJ). Material for the study was gathered from four sites located in three provinces representing north-eastern Poland. The two of them were located in the Podlaskie voivodeship, one in the Mazursko-Warmińskie voivodeship and one in the Mazowieckie voivodeship (Fig. 1). The study sampling areas are a region of low industrialization with many forests, peatlands, and river valleys, which serve as refuges for numerous species of the *Cervidae* family, including elk.

The Minister’s permit primarily covered 90 individuals (30 males and 60 females) to be shot in October and November 2010 for scientific purposes following application of Biology Institute in Bialystok. After protests of environmental NGOs, the permit was withdrawn, and finally, the samples of organs were taken from 5 males and 30 females. Mean age of animals was 7 years, body weight ranged from 68 to 235 kg with a mean of 171 kg.

The animals were divided into age groups. The first group comprised animals less than 2 years old, the second group was composed of fully matured animals older than 3 years. Liver samples had a form of triangles collected from the verge of the right lobe. Kidney samples were collected in a way to contain only the cortex of this organ while muscle samples consisted of the sections of *m. masseter*. After collection, the samples were placed in tight polyethylene bags and frozen at −20 °C. Before the beginning of chemical analyses tissues were homogenized and then 0.5 g of the sample was placed in Teflon containers and mineralized in a microwave apparatus under pressure (system Milestone MSL 1200) in the presence of 7 mL of concentrated nitric acid and in 1 mL of 30 % hydrogen peroxide (Merck, Germany).

Cu and Mo contents were determined with the inductively coupled plasma mass spectrometry (ICP-MS, ELAN DRC II, Perkin Elmer, USA). The method was tested with certified reference material CRM – BCR 185 R (Community Bureau of Reference, BCR in Brussels, Belgium). The percentages of recovery were 118 and 102 % for Cu and Mo, respectively. Discrepancies between the certified values and concentrations quantified were below 10 %. All analyses were performed in triplicate. Obtained results are presented as means expressed in mg∙kg^−1^ wet weight (w.w.) of studied organs.

Statistical analysis was performed with Statistica 10.0 software (StatSoft Inc.). Before analyses, the data were tested for normality with Shapiro-Wilk *W* test. Concentrations of both metals were not normally distributed. Therefore, non-parametric Mann–Whitney *U* test was used to check the significance of differences between age groups. Differences were considered as significant at the level *p* ≤ 0.05. Relationships between the concentrations of copper and molybdenum were calculated using Spearman’s correlation coefficients at *p* ≤ 0.05 and *p* ≤ 0.01.

## Results

Concentrations of Cu and Mo in the liver, kidneys, and muscles of studied animals are presented in Tables 1 and 2. The copper concentrations in the liver, kidneys, and muscles varied markedly within the limits from 1.16 to 87.42, 2.58 to 32.51, and 1.12 to 3.80 mg∙kg^−1^ of the organ (w.w.), respectively. In all study animals, the hepatic Mo concentration varied within the ranges from 0.06 to 1.84 mg∙kg^−1^. In the kidneys and muscles, these values were from 0.04 to 3.63 and 0.03 to 0.25 mg∙kg^−1^, respectively. Age did not significantly affect the content of both metals in analyzed organs. Taking into account the mean Cu and Mo concentrations, the highest values were noted in the liver. A decidedly lower level of both metals was noted in kidneys. Muscles were characterized by the lowest mean values of the examined elements.

**Table 1:** The mean concentrations of copper and molybdenum in liver, kidney, and muscles of the elk

		Cu (mg∙kg^−1^ wet weight)	Mo (mg∙kg^−1^ wet weight)
Liver *n* = 35	Mean ± SD	23.08 ± 27.6	0.92 ± 0.46
	Range	1.16–87.42	0.06–1.84
	Median	8.81	0.89
	Lower quartile (25 %)	3.38	0.64
	Upper quartile (75 %)	35.32	1.23
Kidney *n* = 35	Mean ± SD	5.03 ± 5.29	0.42 ± 0.62
	Range	2.58–32.51	0.04–3.63
	Median	3.93	0.35
	Lower quartile (25 %)	3.51	0.16
	Upper quartile (75 %)	4.41	0.43
Muscle *n* = 35	Mean ± SD	2.36 ± 0.52	0.05 ± 0.03
	Range	1.12–3.80	0.03–0.25
	Median	2.35	0.05
	Lower quartile (25 %)	2.08	0.04
	Upper quartile (75 %)	2.63	0.05

**Table 2:** The mean copper and molybdenum concentrations in the organs of animals depending on age (in mg∙kg^−1^ wet weight)

	Organs	*n*	Elks up to 2 years		*n*	Elks older than 3 years	
			Mean ± SD	Median		Mean ± SD	Median
Cu	Liver	8	18.61 ± 18.63	7.02	27	24.45 ± 30.03	10.77
	Kidney	8	4.51 ± 2.43	3.72	27	5.13 ± 5.73	4.01
	Muscle	8	2.68 ± 0.57	2.44	27	2.28 ± 0.49	2.31
Mo	Liver	8	0.75 ± 0.42	0.79	27	0.97 ± 0.48	1.00
	Kidney	8	0.25 ± 0.33	0.12	27	0.46 ± 0.67	0.35
	Muscle	8	0.07 ± 0.06	0.05	27	0.047 ± 0.02	0.05

In the analysis of correlation between the Cu and Mo levels in particular organs, the presence of significant dependence (*p* ≤ 0.05) was observed in the liver of animals studied. Relationships between the concentrations of analyzed metals in particular organs are given in Table 3.

**Table 3:** Correlation coefficients between copper and molybdenum in the examined organs

Organs		Cu			Mo	
Cu		Liver	Kidneys	Muscles	Liver	Kidneys
	Kidneys	−0.1729				
	Muscles	0.1669	0.0304			
Mo	Liver	0.3824^a^	−0.2082	−0.0890		
	Kidneys	−0.1553	0.3296	−0.0212	0.2086	
	Muscles	0.3380	−0.0166	0.2579	−0.0054	−0.1598

^a^Statistically significant coefficient of correlation at *p* ≤ 0.05

## Discussion

Most studies devoted to mineral composition of elk tissues is based on material from a small number of individuals. This is probably a result of problems with the access to numerous and representative study material. In this context, studies from Sweden are exceptional being based on a great number of individuals and accompanied by the analysis of biochemical blood parameters, cytochrome oxidase activity in myocardium, and because of studied biogeochemistry of selected elements [3, 6, 7, 10]. Particular attention was paid to the content of Cu and Mo because of the so-called “mysterious disease” of unknown etiology that appeared in Sweden in the mid-1980s [7, 11]. Animals with disorders were mainly noted in one region of the country though sick animals were also seen in other regions of Sweden. Viral infection was suspected as the reason of this disease, but the presence of virus was not found during examination of sick animals. Clinical symptoms and changes in organs observed in post-mortem analyses resembled the symptoms noted during copper deficiency and molybdenosis in cattle and sheep [6, 7]. Long-term studies showed that since 1982 till 1994, the content of Cu in liver of individuals without disease symptoms decreased by half, and at the same time, the concentration of molybdenum increased by 20 to 40 % [6, 7, 12, 13]. Based on the results of microbiological and anatomopathological studies and mainly on chemical analyses of elk organs, it was finally concluded that the most probable reason of the disease was Cu deficiency and molybdenosis [6, 7, 11]. The latter hypothesis was supported by finding diabetes type 2 in sick individuals, in which long hyperglycemia resulted in the glycation of proteins [10]. Cu deficits were also described in other species of wild ruminants representing *Cervidae* family like sika deer (*Cervus nippon* Temnick) and red deer (*Cervus elaphus*). Most often, they manifested themselves as neurological disorders in a form of enzootic ataxia [14–16]. Described symptoms result from the fact that Cu is an element determining proper course of metabolic processes, like e.g., cell respiration, activity of nervous system (copper participates in the formation of neurotransmitters), building of connective tissues, and production of pigments in tissues [7, 17, 18]. Biological role of Cu is a result of its properties as a component or activator of enzymes such as peroxide dismutase, ceruloplasmine, cytochrome oxidase, tyrosinase, dopamine hydroxylase, and lysine oxidase. The element plays also a key role in iron metabolism. Copper concentration in adult mammals is from 1.5 to 2.5 mg∙kg^−1^ body mass [19, 20]. Due to specific anatomical features of digestive system in ruminants, there is an interaction between copper and antagonists, particularly molybdenum and sulfur, which in the rumen results in the production of thiomolybdates [18]. An important issue is the demand of rumen microflora for Cu which is lower than the demand of host’s tissues and amounts 1.57 μmol∙kg^−1^ of dry mass of fodder. Respective value for molybdenum is much higher and ranges from 104.2 μmol∙kg^−1^ to 2.85 mmol∙kg^−1^ dry mass of fodder. Proper Mo to Cu ratio in the diet of ruminants is assumed as 1:6–10 [21]. In the case of Cu deficiency, carbohydrate metabolism is disturbed, which leads to disturbances in fat synthesis due to decreased activity of Cu-containing cytochrome c oxidase (COX) [10]. Copper deficiency may occur in a primary form as a lack of copper in animals’ diet or in a secondary form resulting from Mo excess in food [7]. Recognition of Cu deficiency is difficult because clinical symptoms are non-specific and not all appear in each suffering individual [22]. No effective method of estimating organism’s supply with copper in living animals has been elaborated so far [6]. The liver is the main storage organ for Cu and is recommended as a useful indicator of its status in animals [23]. Molybdenum is a component of numerous metalloenzymes. It is known that the metal cooperates with copper and sulfates in many metabolic processes. In low concentrations, it is present in all fluids and tissues of an organism [1, 9, 24, 25]. The highest concentrations of Mo are found in kidneys, liver, small intestine, and in adrenal glands. Ruminants feeding on fodder rich in Mo are exposed to Mo intoxication. Molybdenum toxicity in animals is commonly named molybdenosis and results in Cu deficiency and inappropriate level of sulfates, which markedly affects the relationships between the two metals [26–28].

Frank et al. [7] studying animals from Sweden found that the mean concentration of Cu in the liver of healthy individuals (control group) was 29.0 mg∙kg^−1^ w.w. and in sick individuals, 11.4 mg∙kg^−1^. No statistically significant differences between groups were found because Cu concentrations varied markedly ranging from 3.93 to 106 mg∙kg^−1^ in healthy animals and from 3.2 to 28.2 mg∙kg^−1^ in sick ones. Our results were similar to those found in healthy animals from Sweden. The concentration of Cu varied from 1.16 to 87.42 mg∙kg^−1^with the mean of 23.08 mg∙kg^−1^. Molybdenum concentration in the liver of healthy individuals from Sweden was 0.855 mg∙kg^−1^ on average and in sick animals from the same region 1.165 mg∙kg^−1^. Studied groups differed significantly in Mo concentration. In animals from north-eastern Poland, mean concentration of Mo was 0.92 mg∙kg^−1^ with a minimum of 0.06 and maximum 1.84 mg∙kg^−1^. Noteworthy, according to hunters, none of the animals shot in Poland showed disease symptoms similar to those observed in Swedish elks. The concentration of Cu in the kidneys of healthy individuals from Sweden was 3.8 mg∙kg^−1^ (w.w.) on average while in sick individuals, the respective value was 5.469 mg∙kg^−1^ being similar to results obtained in the analysis of elks from Poland (5.03 mg∙kg^−1^). In other studies published by Frank et al. [8], median concentration of Cu and Mo in elk’s liver was 34 and 0.82 mg∙kg^−1^, respectively. In our study, the respective medians were 8.81 and 0.89 mg∙kg^−1^. A decrease of Cu content was also noted in other organs of sick animals. Frank et al. [10] found decreased Cu content in heart muscle of animals suffering from “mysterious disease”. In a group of healthy animals, Cu concentration was 3.79 mg∙kg^−1^ fresh weight and in sick ones, 3.02 mg∙kg^−1^. Reverse relationship was found for Mo whose concentration in myocardium of healthy animals was 0.013 while in sick animals, it amounted 0.031 mg∙kg^−1^.

Results of studies of 25 individuals of elk from Karelia were higher than ours and those obtained by Swedish authors [29]. Mean content of Cu in liver was 43.54 mg∙kg^−1^w.w., in muscles 3.80, and in myocardium 6.38 mg∙kg^−1^ (w.w.). The concentration of Cu in the liver of Russian elks was nearly twice that in animals from north-eastern Poland.

Gamberg et al. [30] found 40.32, 3.47, and 1.48 mg∙kg^−1^ w.w. of Cu in the liver, kidneys, and muscles, respectively, of elk from Yukon. Copper content in the liver of Alaskan elks was definitely higher than that obtained in our studies. Elks from Poland showed higher Cu content in other studied organs. Concentrations of molybdenum in liver were almost identical in both populations—0.90 mg∙kg^−1^ in elks from Yukon and 0.91 mg∙kg^−1^ in Polish elks. In kidneys and muscles, the relations were similar to those observed for Cu. In American elks, the concentrations of Mo were 0.27 and 0.01 mg∙kg^−1^, respectively. Frank et al. [3] who analyzed tissues of elks from Nova Scotia that suffered from “mysterious disease” with symptoms and anatomopathological changes similar to those in Swedish animals found the median content of copper in the liver, kidneys, and heart muscle equal 50.0, 3.7, and 3.15 mg∙kg^−1^ (w.w.), respectively. Respective concentrations of Mo were 0.600, 0.199, and 0.0055 mg∙kg^−1^. Median content of Cu in the liver of Polish elks was clearly lower while concentrations found in kidneys and muscles were similar. Median content of Mo in analyzed organs was higher in Polish elks.

Comparing the results obtained to the data concerning Cu concentrations in the muscles of other large free-living ruminants inhabiting north-eastern Poland, such as the red deer, it can be stated that these values are roughly within the ranges noted by Falandysz et al. [31] (mean values from 1.9 to 6.4 mg∙kg^1^ w.w.), Jarzyńska i Falandysz [1] (median value 3.3 mg∙kg^−1^ w.w.), and Skibniewski et al. [2] (median value 2.79 mg∙kg^−1^ w.w.).

Low median values of Cu content in liver, which is the main store of this metal, in comparison with Swedish and North American studies may indicate inappropriate supply of Cu in elks from north-eastern Poland. Deficit of this element may thus be observed without visible clinical symptoms. Low content of Cu in soils of this region may contribute to this phenomenon. Studies of arable soils in Poland performed in years 1995–2010 showed low Cu concentrations in Podlaskie voivodeship. In the control point adjacent to the habitats of the examined animals, the mean Cu concentration in 1995 was 3.8, while in 2010 3.4 mg∙kg^−1^ [32]. Particularly poor in Cu are river valleys, acid meadows, and peatlands [27]. Such areas are typical refuges of elk in north-eastern Poland. A lack of statistically significant differences between age groups is due to variability of copper concentrations in analyzed samples. Higher concentrations were, however, observed in the liver and kidneys of older animals. This is a phenomenon similar to that observed in mammals of other taxonomic groups in which Cu tends to accumulate in liver and kidneys with age [18]. The reverse relationship was found in muscles where higher concentrations of both metals were found in young individuals.

It is difficult to explain statistically significant positive correlation between the content of Cu and Mo in the liver of analyzed animals since both elements are in an antagonistic relationship as a result of their electron configuration in ionic form [20]. The explanation of this phenomenon may be homeostatic mechanisms allowing storage of both metals in the liver following their absorption in the gastrointestinal tract. Low dietary levels of Cu and Mo, which are absorbed via venous portal system within physiological ranges, do not cause strong interaction between them. Although Cu concentration in the liver of the animals studied was low, they probably developed evolutionary mechanisms allowing them to tolerate hypocupremia in periods of scarcity, similarly to a phenomenon which has been described in other species representing free living and domesticated ruminants [33].

## Conclusions

Summing up, it can be stated that the content of Cu in the liver of elks from north-eastern Poland is lower compared with data reported from Sweden, Russia, and North America. Concentrations of Cu and Mo in the kidneys and skeletal muscles of Polish elks are similar to data noted in healthy animals from Sweden. No statistically significant differences were observed between age groups. Obtained results suggest that animals from this region may be threatened by primary Cu deficiency but do not allow for conclusions of normative character pertaining to the whole elk population. We are of the opinion that studies on mineral supply of elks in Poland should be continued to produce a reference base of mineral components in organs of this species. Such a database would allow for the assessment of mineral supply of animals and for estimating potential risks associated with the disturbance of mineral balance.
